# Post-traumatic stress disorder and major depression in conflict-affected populations: an epidemiological model and predictor analysis

**DOI:** 10.1017/gmh.2015.26

**Published:** 2016-02-10

**Authors:** F. J. Charlson, A. Flaxman, A. J. Ferrari, T. Vos, Z. Steel, H. A. Whiteford

**Affiliations:** 1 University of Queensland, School of Public Health, Herston, QLD, Australia; 2 Queensland Centre for Mental Health Research, Wacol, QLD, Australia; 3 University of Washington, Institute for Health Metrics and Evaluation, Seattle, USA; 4 University of Sydney, School of Psychiatry, Sydney, NSW, Australia; 5 St John of God, Richmond Hospital, North Richmond, NSW, Australia

**Keywords:** Aetiology, conflict, depression, epidemiology, global mental health, post-traumatic stress disorder

## Abstract

**Background:**

Despite significant research examining mental health in conflict-affected populations we do not yet have a comprehensive epidemiological model of how mental disorders are distributed, or which factors influence the epidemiology in these populations. We aim to derive prevalence estimates specific for region, age and sex of major depression, and PTSD in the general populations of areas exposed to conflict, whilst controlling for an extensive range of covariates.

**Methods:**

A systematic review was conducted to identify epidemiological estimates of depression and PTSD in conflict-affected populations and potential predictors. We analyse data using Bayesian meta-regression techniques.

**Results:**

We identified 83 studies and a list of 34 potential predictors. The age-standardised pooled prevalence of PTSD was 12.9% (95% UI 6.9–22.9), and major depression 7.6% (95% UI 5.1–10.9) – markedly lower than estimated in previous research but over two-times higher than the mean prevalence estimated by the Global Burden of Disease Study [3.7% (95% UI 3.0–4.5) and 3.5% (95% UI 2.9–4.2) for anxiety disorders and MDD, respectively]. The age-patterns reveal sharp prevalence inclines in the childhood years. A number of ecological variables demonstrated associations with prevalence of both disorders. Symptom scales were shown to significantly overestimate prevalence of both disorders. Finding suggests higher prevalence of both disorders in females.

**Conclusion:**

This study provides, for the first time, age-specific estimates of PTSD and depression prevalence adjusted for an extensive range of covariates and is a significant advancement on our current understanding of the epidemiology in conflict-affected populations.

## Introduction

The significant association between war-trauma exposure and elevated mental disorder prevalence has been demonstrated repeatedly in epidemiological studies and a causal effect between war-trauma exposure and reduced mental health of a population is generally acknowledged. (Kalt *et al*. 2013; Karam *et al*. 2014) Recent years have seen increased interest and research into the effect of war and conflict on mental disorder epidemiology, resulting in an increase in published material on the topic. The overwhelming majority of the literature has investigated the association between trauma exposure and post-traumatic stress disorder (PTSD). Depression is the only other mental disorder featuring prominently in the literature, in this context. Despite these advances we do not yet have a comprehensive epidemiological profile (including prevalence, distribution and risk factors) of PTSD or depression in conflict-affected populations, which is essential for the development of effective mental health policy and programmes addressing the mental health needs of these populations.

In order to meet the challenges of post-conflict reconstruction, comprehensive and context-specific epidemiological models of mental disorders are crucial for both conflict-affected countries and the broader international health and humanitarian community. They provide the core data required for estimating disease prevalence, i.e. the number of cases of a given disease within conflict-affected populations at a particular point in time. This, when supplemented with a variety of adjunct data sources, forms the basis for estimating mental health service requirements during the post-conflict period and for comparisons with current service capacity (Bruckner *et al*. 2011; World Health Organization, 2011). Furthermore, prevalence data are an essential input to the estimation of years lived with disability – the non-fatal component of burden of disease estimates (Vos *et al.* 2012). These estimates of non-fatal disease burden can be used in conjunction with United Nation's population data (United Nations, 2011) to project changes in the burden of mental and substance use disorders during and after conflict and, subsequently, predict the mental health workforce required to meet future needs (Charlson *et al*. 2012, 2014).

Although several published papers review the epidemiology of mental disorders in conflict-affected populations, only two studies have conducted pooled analyses of surveys representative of the general population – one limited to examining prevalence of mental disorders among children exposed to war (Attanayake *et al*. 2009); and another limited to the same in the adult population (Steel *et al*. 2009). Quantitative assessment of predictors for mental disorder prevalence in conflict-affected populations has also been attempted previously (Porter & Haslam, 2005; Steel *et al*. 2009); however, significant methodological limitations were present. To-date, epidemiological modelling of mental disorder prevalence in conflict-affected populations across the different ages, and which incorporates an extensive range of covariates using advanced meta-regression techniques, has not yet been undertaken.

The Global Burden of Disease Study 2013 (GBD2013) provides the most recent and comprehensive epidemiological profile for a variety of mental disorders (Global Burden of Disease Study 2013 Collaborators, 2015). Prevalence estimates have been calculated at the region and country level and provide a useful benchmark for which to compare estimates from conflict-affected subpopulations (Ferrari *et al*. 2013; Baxter *et al*. 2014). In addition, the GBD methodology has developed a Bayesian meta-regression tool that can be drawn upon to develop robust epidemiological models, which are also able to explore the effect of predictor variables on prevalence (Flaxman *et al*. 2013).

This paper aims to derive a comprehensive epidemiological profile of major depression and PTSD in general (civilian) populations exposed to conflict by: (1) conducting a systematic review to identify studies investigating the epidemiology of major depression and PTSD in conflict-affected populations across different age groups; (2) identifying significant predictors of the prevalence of major depression and PTSD in conflict-affected populations; and (3) making use of the above data and GBD Bayesian meta-regression techniques to model the prevalence of major depression and PTSD by age, sex and region.

## Methods

### Systematic review

A systematic review of the literature was conducted following PRISMA guidelines (Liberati *et al*. 2009). A systematic search of electronic databases and grey literature sources identified data sources for the prevalence of major depression and PTSD cases which met criteria as per the Diagnostic and Statistical Manual of Mental disorders (DSM) or the International Classification of Diseases (ICD) (World Health Organization, 1992; American Psychiatric Association, 2000). Additional detail of the systematic review can be found in the Online appendix.

### Inclusion and exclusion criteria

Inclusion criteria have been imposed on study selection requiring: (1) study samples be representative of the general conflict-affected population; (2) participants be situated in the country of origin, displaced, or resettled in another non-Western country; (3) studies report epidemiological estimates from either cross-sectional or longitudinal population-based surveys, (4) survey instruments map to DSM or ICD criteria; and (5) data be for the period 1980 onward. Study samples were excluded if they were seeking asylum or resettled in western countries, combatants (including child soldiers), family members of combatants, clinical samples, student samples, exposed to only isolated terrorist attacks (such as 9/11), torture victims, health workers, ex-prisoners of war or political detainees, and offender samples. Additional detail of the inclusion and exclusion criteria can be found in the appendix.

### Statistical methods

One way in which to make important advances on current predictive modelling efforts is to develop epidemiological estimates specific to the country/region, age and sex, whilst adjusting for a range of covariates responsible for any heterogeneity within a dataset. Information pertaining to the epidemiological data surveyed and the survey methodology were extracted from each study. Additionally, a list of variables previously shown to have significant associations with mental disorder prevalence was identified from the literature. This included ecological variables which hold relationships with health and/or mental health extracted from online databases including the United Nations, World Bank, and GBD resources. A full list of variables explored can be found in the appendix.

Analysis was conducted in two stages. At stage one, we followed previously established GBD methodology for summarising and predicting epidemiological data while adjusting for heterogeneity between studies (Charlson *et al*. 2013; Ferrari *et al*. 2013). All relevant variables were initially assessed for significant associations with prevalence in univariate analyses in Stata 11 (StataCorp, 2009). For each disorder respectively, statistically significant variables (from univariate analyses) were included in a linear regression model with prevalence as the dependent variable. Independent variables were systematically added and removed based on their contribution and influence on prevalence. This was assessed by an overall adjusted *R*^2^, and coefficient and *p* value derived for each variable. The final decision on whether a variable was to be included as a covariate in the stage two modelling was dependent on these findings and/or whether the variable was considered to be integral to the core research questions (e.g. sex and world region).

At stage two, we made use of DisMod-MR, a Bayesian meta-regression tool designed specifically for GBD purposes (Vos *et al*. 2012; Flaxman *et al*. 2013). DisMod-MR is used to estimate age–sex–country-specific prevalence from heterogeneous and often sparse datasets such as in this study. DisMod-MR makes use of a negative-binomial model and fits models using a randomised Markov-Chain Monte Carlo algorithm. Further details on DisMod-MR and its assumptions can be found in the appendix of this paper and in the online supplementary material of Vos *et al*. 2013 (Vos *et al*. 2012) and Flaxman *et al*. 2014 (Flaxman *et al*. 2013).

## Results

### Systematic review

**Fig. 1.**
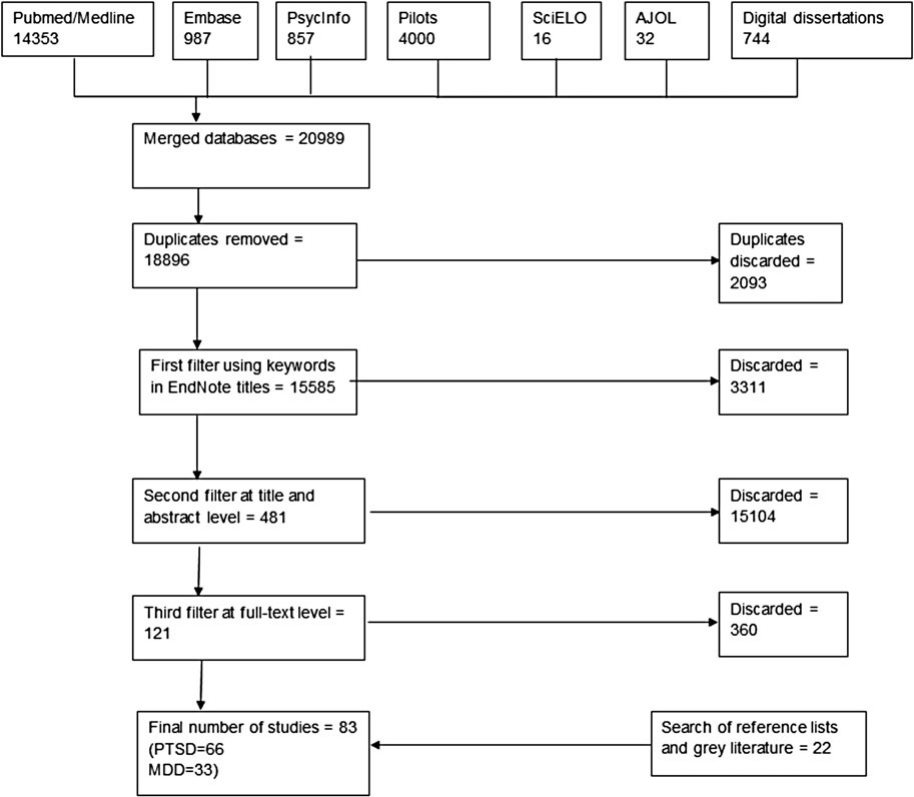
Search flow diagram.

We identified a total of 83 studies; 66 studies providing 128 prevalence estimates for PTSD and 33 studies providing 69 prevalence estimates for major depression. Twenty separate conflict or post-conflict countries were represented for major depression and 27 conflict or post-conflict countries for PTSD. A summary of the search flow diagram and included studies can be found in Figure 1.

### Identifying significant covariates

Several potential predictors of prevalence were identified, and tested for univariate associations. Covariates assessed, *p* values from correlation tests and the final model inclusion/exclusion status based on the decision-making process outlined in the methods can be found in Table 1. A full description of covariates along with reasons for exclusion from models can be found in the appendix.

**Table 1.** Covariate correlation test p values and final model inclusion/exclusion status

	PTSD		Major depression	
Covariate name	Statistical significance of correlation test	Status	Statistical significance of correlation test	Status
*Study-level covariates*				
Study type	*p* = 0.169	Excluded	*p* = 0.492	Excluded
Coverage	*p* = 0.005	Included	*p* = 0.701	Excluded
Population description	*p* = 0.328	Excluded	N/A	N/A
Education	*p* = 0.619	Excluded	*p* = 0.895	Excluded
Marital status	*p* = 0.439	Excluded	*p* = 0.292	Excluded
Diagnostic interview	*p* = 0.104	Included	*p* = 0.000	Included
Response rate	*p* = 0.001	Included	*p* = 0.004	Included
Urbanicity	*p* = 0.335	Excluded	*p* = 0.264	Excluded
Sex	*p* = 0.008	Included	*p* = 0.050	Included
Adults or children	*p* = 0.427	Excluded	*p* = 0.864	Excluded
Type of estimate	*p* = 0.000	Excluded	*p* = 0.010	Excluded
*Trauma covariates*				
Political terror scale rating	*p* = 0.230	Excluded	*p* = 0.009	Excluded
War-related events ratio	*p* = 0.001	Included	*p* = 0.913	Excluded
*Country-level covariates*				
World region	*p* = 0.049	Included	*p* = 0.350	Included
Time since conflict	*p* = 0.069	Included	*p* = 0.104	Included
Time since max PTS	*p* = 0.054	Excluded	*p* = 0.000	Excluded
Length of conflict	*p* = 0.675	Excluded	*p* = 0.049	Excluded
Alcohol usage	*p* = 0.094	Excluded	*p* = 0.115	Excluded
Drug usage	*p* = 0.000	Excluded	*p* = 0.003	Excluded

Note: The war-related events ratio of an individual study is defined as the ratio of ‘the average number of war-related traumatic events with positive responses’ to ‘total number of events screened for’ (this does not include adversity related events which are captured in other variables such as unemployment). For example, if a study screened for 10 war-related events and the average number of positively responded items across subjects was 3, then the war-related events ratio for that particular study is 0.3.

Note: Covariates representing the social and economic conditions within a country excluded from modelling due to interactions with time are not included in Table 1.

Univariate tests confirmed some anticipated associations including an apparent sex difference in prevalence for both disorders, differences in prevalence as measured by diagnostic *v*. symptom-based instruments for both disorders, and an effect of trauma exposure on PTSD (as measured by the war-related events ratio). Notably, there was an association between the war-related events ratio and PTSD prevalence but not prevalence of major depression.

Assessment of country-level covariates showed a number of statistically significant associations. A large number of covariates representing the social and economic conditions within a country were associated with prevalence of both disorders (e.g. education index, life expectancy, and illicit drug use; see appendix for a full list). However, statistical testing confirmed the hypothesis that these variables and prevalence are likely to all vary with time. This interaction with time enhances the risk of apparent associations which are spurious and these country-level covariates were therefore excluded from modelling (Altman *et al*. 1983).

A covariate representing the number of years passed since the maximum rating on the Political Terror Scale for a country (time since max PTS) was used as an alternative measure of time since the end of conflict. Univariate tests showed that this covariate was strongly associated with major depression but not PTSD. However, when entered into a regression model ‘time since max PTS’ no longer contributed significantly to the prevalence of major depression. Time since conflict was a more influential covariate for both PTSD and major depression than time since max PTS and was included in the final model for both disorders.

Table 2 shows the relative risk (RR) of each of the included covariates as an output of DisMod-MR prevalence models. Relative trauma exposure from war-related events as measured by the ‘war-related events ratio’ demonstrated that study populations experiencing a ratio greater than 0.3 were 1.2 times at greater risk of PTSD than those with lower levels of conflict exposure, although this effect was not statistically significant (95% UI 0.9–1.6). Response rate did not have a statistically significant effect on prevalence. Symptom scales were shown to significantly overestimate prevalence of both PTSD and major depression. Differences across world regions or the post-conflict period were not evident.

**Table 2.** Relative risks (RR) of covariates estimated from DisMod-MR modelling

PTSD	RR (95% UI)	Major depression	RR (95% UI)
War-related events ratio		Instrument type	
<0.3	1.0	Diagnostic interview	1.0
>0.3	1.2 (0.9–1.6)	Symptom scale	2.6 (1.7–4.0)*
Instrument type		Response rate	
Diagnostic interview	1.0	>80%	1.0
Symptom scale	1.7 (1.1–2.5)*	<80%	0.9 (0.5–1.5)
Coverage		Sex	
National	1.0	Females	1.0
Regional	1.1 (0.7–1.6)	Males	0.6 (0.4–1.0)
Community	1.7 (0.7–1.6)	Time since conflict	
Response rate		0 (still in conflict)	1.0
>80%	1.0	1–2 years	1.6 (0.8–3.1)
50–79%	0.6 (0.4–0.9)	3–4 years	0.6 (0.4–1.1)
Sex		5–7 years	0.4 (0.2–1.1)
Females	1.0	8–10 years	1.1 (0.5–2.9)
Males	0.7 (0.5–1.0)	World region	
Time since conflict		North Africa/Middle East	1.1 (0.9–1.3)
0 (still in conflict)	1.0	Southeast Asia	1.0 (0.8–1.1)
1–2 years	0.8 (0.5–1.1)	Central Europe	1.0 (0.9–1.3)
3–4 years	1.1 (0.7–1.7)	High-income countries	1.0 (0.8–1.2)
5–7 years	1.3 (0.8–2.3)	Latin America	1.0 (0.8–1.1)
8–10 years	0.8 (0.5–1.2)	Sub-Saharan Africa	1.0 (0.8–1.1)
World region		South Asia	1.0 (0.8–1.1)
North Africa/Middle East	1.1 (0.9–1.4)		
Southeast Asia	0.9 (0.7–1.1)		
Central Europe	1.1 (0.9–1.4)		
High-income countries	0.9 (0.7–1.1)		
Latin America	1.0 (0.8–1.2)		
Sub-Saharan Africa	1.2 (1.0–1.5)		
South Asia	0.9 (0.7–1.1)		

*Statistically significant RR.

### DisMod-MR prevalence modelling

Unadjusted pooled prevalence was found to be 30.0% (95% UI 26.1–33.9) for PTSD and 20.2% (95% UI 15.0–25.8) for major depression. Making adjustments to prevalence using the covariates listed, DisMod-MR estimated the age-standardised pooled prevalence of PTSD for conflict-affected populations in our dataset at 12.9% (95% UI 6.9–22.9) and for major depression at 7.6% (95% UI 5.1–10.9). The latter is compared with a mean major depressive disorder (MDD) global prevalence of 3.5% (95% UI 2.9–4.2) estimated in GBD2013 (Institute of Health Metrics and Evaluation, 2015).

**Fig. 2.**
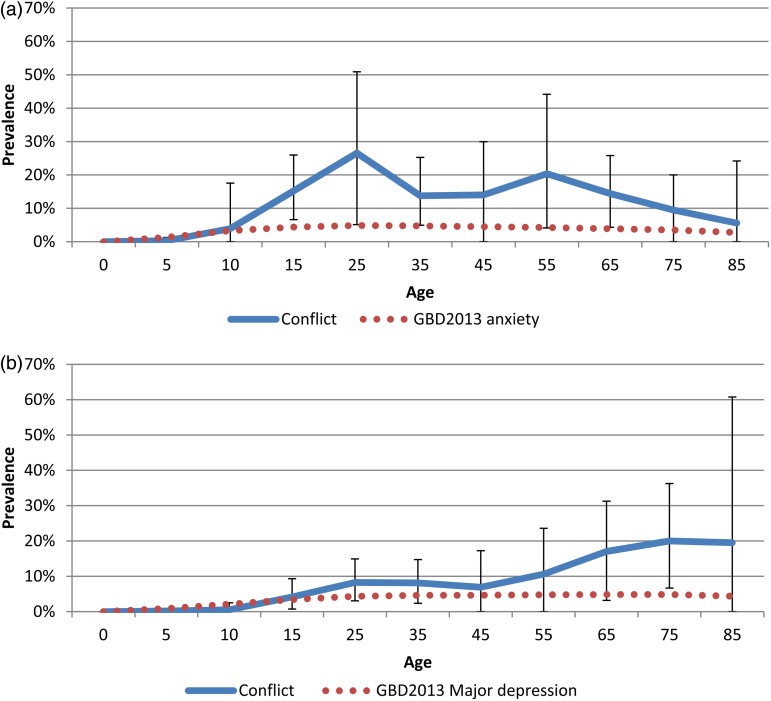
(*a*) Age-specific prevalence of PTSD in conflict-affected populations (95% UI). (*b*) Age-specific prevalence of major depression in conflict-affected populations (95% UI). Note: GBD2013 modelled the prevalence of all anxiety disorders combined rather than PTSD specifically.

Figure 2*a* shows the modelled age-specific PTSD prevalence in conflict-affected populations benchmarked against the GBD2013 mean global prevalence of all anxiety disorders (Institute of Health Metrics and Evaluation, 2015). PTSD prevalence revealed a sharp incline in prevalence in childhood years peaking at around 25 years and a decline after 55 years. The age-specific prevalence for all anxiety disorders as estimated in GBD2013 was lower at all ages. Figure 2*b* shows the modelled age-specific major depression prevalence in conflict-affected populations benchmarked against the GBD2013 mean global prevalence of MDD. There was a steady upward trend in prevalence peaking around age 20. The second peak in the elderly is high but surrounded by overlapping bounds of uncertainty due to scarcity of data. It is important to note the ranges of uncertainty surrounding estimates when interpreting these findings.

The data suggest a higher prevalence of both PTSD and major depression in females; however, this finding was not statistically significant (Table 2). There were no reported differences in exposure to war-trauma between males and females as assessed by the number of categories of war-related potentially traumatic events endorsed (not shown). No statistically significant variations in prevalence of either major depression or PTSD were found across conflict-affected populations stratified by region. However, comparison of the estimated mean major depression prevalence in conflict-affected populations with GBD2013 MDD estimates for countries contributing to our dataset, demonstrates that GBD estimates are lower than our estimated prevalence for several conflict-affected countries (Table 3).

**Table 3.** Analysis of GBD 2013 country-specific MDD prevalence estimates for conflict-affected countries, 2013

Country	GBD 2013 prevalence estimate (%, 95% uncertainty)	Difference from mean for conflict-affected populations^a^
Bosnia-Herzegovina	3.1 (2.7–3.6)	Lower
Mexico	4.5 (2.8–6.1)	Not statistically different
Afghanistan	3.4 (1.6–5.3)	Not statistically different
Iraq	3.1 (2.3–3.9)	Lower
Lebanon	3.5 (2.9–4.1)	Lower
Palestine	7.7 (6.1–9.5)	Not statistically different
Nepal	2.5 (1.8–3.5)	Lower
Pakistan	4.5 (3.1–6.2)	Not statistically different
Indonesia	2.2 (1.4–2.9)	Lower
Sri Lanka	2.3 (1.5–3.0)	Lower
Timor-Leste	1.9 (1.3–2.6)	Lower
Central African Republic	5.0 (3.6–6.3)	Not statistically different
Democratic Republic of Congo	5.1 (3.8–6.4)	Not statistically different
Ethiopia	5.4 (4.4–6.7)	Not statistically different
Liberia	4.0 (2.9–5.2)	Not statistically different
Nigeria	4.4 (3.4–5.5)	Not statistically different
Rwanda	6.5 (5.0–8.1)	Not statistically different
South Africa	4.0 (3.3–4.8)	Lower
Sudan	4.5 (3.4–6.0)	Not statistically different
Uganda	6.9 (5.6–8.5)	Not statistically different
Israel	3.1 (2.1–4.4)	Lower
Northern Ireland	3.1 (2.7–3.6)	Lower
Cambodia	2.2 (1.4–3.0)	Lower
Croatia	3.1 (2.7–3.5)	Lower
Iran	4.5 (2.7–6.3)	Not statistically different
Mozambique	5.8 (4.3–7.5)	Not statistically different
Peru	4.0 (3.1–4.8)	Lower
Somalia	6.0 (4.4–7.6)	Not statistically different
Turkey	3.2 (2.8–3.5)	Lower

a Mean major depression prevalence for conflict-affected populations is 7.6% (95% UI 5.1–10.9).

Heterogeneity in our datasets was large and the median value of the negative binomial model overdispersion parameter calculated by DisMod-MR for PTSD was 2.5 (where zero is completely uninformative, and infinity is a Poisson distribution). The equivalent for major depression was 1.9. Sensitivity analyses looking for outliers in both datasets were conducted by removing one input prevalence estimate at a time during meta-regression analyses to see if they significantly impacted upon results. Apparent outliers were deemed to have been sufficiently adjusted for by covariates in the meta-regression and there were no estimates removed from analyses as a result.

## Discussion

This paper builds upon current knowledge, providing for the first time, age-specific prevalence estimates of major depression and PTSD in the general (civilian) populations of areas exposed to conflict within the past 10 years. It differentially assesses the population in conflict-affected areas who reside in the conflict region or have been displaced to neighbouring regions (not those resettled in Western countries) and is all-ages inclusive. The inclusion criteria are more stringent than that of previous literature in order to ensure optimum representativeness and reduced heterogeneity in prevalence estimates. Our review only included studies of participants from predefined conflict or post-conflict settings.

A key objective of this study was to derive findings that can be helpful in anticipating prevalence when a community-representative, diagnostic survey is not feasible – a necessity for informed policy and programming. This paper reiterates the elevated prevalence of major depression and PTSD in conflict-affected populations but estimates markedly lower unadjusted, age-specific and age-standardised prevalence than estimated by previous research of conflict-affected populations (Steel *et al*. 2009). The observed differences seen between the current study and previous work are largely due to more stringent inclusion and exclusion criteria, optimised search strategies and advances in statistical methods, but also demonstrate the importance of updating systematic reviews over time. Our optimised search strategy is not one that has been used in a systematic review of this topic before and was very effective in identifying additional relevant literature.

Contrasting our predictive estimates against estimates from GBD2013 highlights how relying on global or national estimates of mental disorders can be inherently misleading when dealing with conflict-affected sub-populations. Conflict-affected populations are often geographically constrained to only parts of a country and are typically underrepresented in broader national estimates of prevalence. The modelled prevalence of major depression in conflict-affected populations was over two-times higher than the mean global MDD prevalence estimated in GBD2013. Our findings also point to a highly elevated state of PTSD in conflict-affected populations – more than three times higher than the global mean prevalence of all anxiety disorders combined as estimated by GBD2013 [3.7% (95% UI 3.0–4.5)].

Our analysis of age trends suggested a rise in PTSD prevalence throughout childhood and adolescent years and a relative stabilisation of rates until around 60 years of age. The high rates of PTSD in children is a finding consistent with child-specific reviews (Attanayake *et al*. 2009) and suggests that children show particular sensitivity to developing adverse mental health outcomes following exposure to organised violence and mass conflict. That said, it is also possible that the observed differences in prevalence across ages may be partly driven by the different instruments used in assessing PTSD in children and adults.

In line with GBD2013, our findings for major depression show a peak in prevalence at around 20 years of age, with an increase in prevalence in the older ages. GBD2013 estimates did suggest a smaller increase in the oldest age group compared with our findings however large uncertainty around estimates due to scarcity of data at these ages makes parallel findings in conflict-affected populations difficult to interpret.

Our comprehensive and systematic assessment of potential predictors and selection of model covariates was a valuable aspect of this study and demonstrated a number of important findings. Furthermore, the importance of covariate adjustments in reducing inter study variability within DisMod-MR is demonstrated through the large adjustments made to the data. A covariate designed to adjust symptom scales towards diagnostic interviews found symptom scales to significantly overestimate major depression prevalence – RR 2.6 (95% UI 1.7–4.0). The same analysis for PTSD found a RR of 1.7 (95% UI 1.1–2.5). These findings should provide caution to mental health professionals when utilising prevalence data derived from symptom scales to describe clinically significant presentations of mental disorders. They may also be useful in the interpretation of other study findings by allowing for broad adjustments and/or more meaningful comparisons between estimates derived from symptom scales and those from diagnostic instruments.

In order to reflect the DSM-IV diagnostic criterion of necessarily being exposed to a traumatic event to meet diagnosis for PTSD, and the fact that we are attempting to assess populations affected by war, we elected to create a variable that would measure exposure to traumatic events directly related to war, the ‘war-related events ratio’. The derivation of this ratio drew on previous work by Steel and colleagues who created a variation of this ratio including adverse events not necessarily directly related to war (Steel *et al*. 2009). The odds ratio, derived from a meta-regression model using prevalence as the dependant variable, for Steel and colleagues’ covariate was greater than 3 for a ratio greater than 0.3. In our PTSD model, the war-related events ratio of greater than 0.3 revealed an effect size of only 1.2. This could be due to the more relaxed definition of exposure to ‘adverse’ events by Steel *et al.* more stringent inclusion criteria in terms of accepted populations, or differences in regression methods; however, our finding is likely to represent a more representative effect of exposure to traumatic events directly related to the war itself. Exposure to trauma as measured by the war-related events ratio was not significantly associated to the prevalence of major depression in the current analysis but played a role in PTSD prevalence, confirming findings of previous work (Steel *et al*. 2009; Roberts & Browne, 2011).

All United Nations and World Bank ecological variables (UNDP education index, World Bank GDP per capita, gender inequality index, government effectiveness, United Nations life expectancy, labour participation rate, housing, and improved water source) included in our analyses demonstrated high associations with the prevalence of major depression and PTSD. Unfortunately, they also demonstrated high multi-collinearity with each other and time since conflict hence were necessarily excluded from meta-regression models. The fact that these variables demonstrated high collinearity with time since maximum PTS rating is of interest however and a logical explanation is that as time passes after the end of a period of instability, general social and economic conditions within a country improve. There was also a strong association between major depression prevalence and time since maximum PTS which was not found for PTSD. This lends strength to a theory that major depression prevalence may be more sensitive to the effects of day-to-day challenges (represented by several UN/World Bank ecological variables) than PTSD.

Another potential predictor which is known to be associated with PTSD and major depression prevalence is intimate partner violence (IPV). However, as high quality, representative IPV prevalence data were available only for females the covariate could not be included in our analysis (Devries *et al*. 2013). Nonetheless, its impact on poor mental health should not be overlooked. The higher prevalence of both disorders found in females is consistent with the literature which has proposed the poor mental health of women may include social factors such as domestic and sexual violence which are prominent in conflict contexts (Roberts & Browne, 2011).

A final covariate that we attempted to include in our models is the Hofstede rating on Individualism. This covariate was chosen to reflect a level of social support experienced within a population which has been shown in many studies to act as a protective factor for mental disorders (Roberts & Browne, 2011). The Hofstede theory defines ‘individualist societies’ as those where people are expected to care and provide for themselves and their direct family only. ‘Collectivist’ societies represent a preference for a tightly-knit framework in society in which individuals can expect their relatives or members of a particular in-group to care and provide for them in exchange for unquestioning loyalty (The Hofstede Centre, 2014). This covariate was excluded from modelling due to multicollinearity with time since conflict; however, in univariate analyses it showed a strong association with PTSD in particular. That is, a loosely knit social framework may act as a risk for PTSD and, conversely, a tightly knit society may act as a protective factor for PTSD. More in-depth investigation into the association between this variable and the prevalence of major depression and PTSD is required for clearer conclusions.

Little is known about the natural history of major depression and PTSD over the post-conflict period. Although sparse and heterogeneous data produced non-significant time trends of prevalence, there may be value in creating hypotheses around these findings given what we know about the time course of MDD and PTSD from other research. Across the lifespan, remission rates of PTSD are relatively high; however, even in conflict-free settings in developed nations the median time to remission can be lengthy (Chapman *et al*. 2012). The lack of an observed time trend in our analyses of a 10-year post-conflict period could therefore be reasonable expected. Furthermore, a representative sample with war-related PTSD in five Balkan countries and three Western European countries were assessed on average 8 years after the war and reinterviewed 1 year later. Several years after the war, people with PTSD reported significant symptom improvement that may indicate remission. However, persistent co-morbid depression among refugees was noted (Priebe *et al*. 2012).

Despite an apparent association between disorder prevalence and drug and alcohol use, we were unable to include this in meta-regression models. Given that the literature supports the theory that trauma is associated with a host of adverse behavioural outcomes including substance use, independent analyses assessing alcohol and drug use as mediators of mental disorder prevalence in these populations may be a worthy exercise for future consideration.

### Limitations

The most significant limitation in this study came from the raw data. Even with relatively strict inclusion criteria in place, there was considerable heterogeneity between reported estimates in the major depression and PTSD datasets which restricted analyses to some degree and created large uncertainty around the predicted estimates. This heterogeneity stemmed largely from differences across study designs which are inherently problematic in transcultural and psychiatric epidemiology, particularly following major emergencies (Rodin & Van Ommeren, 2009). Despite this limitation, the strengths of DisMod-MR in dealing with heterogeneity through adjustments to the data (if a significant bias for one or more of the covariates is detected) has allowed us to create an improved epidemiological profile of major depression and PTSD in conflict-affected populations.

A number of assumptions and proxy inputs were used in our estimations given the lack of data available on the epidemiology of major depression and PTSD in conflict-affected populations. Bayesian modelling allows us to integrate all available data on the epidemiology of a given disorder, even when they are sparse and heterogeneous; however, it requires expert-derived assumptions, in the form of ‘priors’, for the model parameters. We strove to limit the use of such expert-derived priors, and instead, to derive a more data-driven prevalence model; however, this led to estimates with large uncertainty intervals. As more and better quality prevalence data are made available, the assumptions made for this analysis can be more adequately tested. Until then, they are limitations which need to be considered when interpreting our findings.

Whilst many sources of variability are common across most fields of epidemiological research, a number of them are particularly intrinsic to mental health research. Variability is added through the use of different instruments used to measure major depression and PTSD. These are available in various versions reflecting the lack of a gold standard, the reliance on Western classification systems to measure major depression and PTSD in developing regions, and continual changes in diagnostic criteria (Whiteford *et al*. 2013). Cultural differences in terms of both culture-specific disorders and the outward expression of disorders create controversy in this respect, particularly within the conflict-affected populations included in this analysis.

Naturally, conducting epidemiological surveys in conflict or post-conflict settings carries its own inherent set of constraints. Limited resources and access to the affected population may result in sub-optimal sampling methods and lower representativeness. Quality of studies was kept at an optimum through the use of strict inclusion criteria, including the prerequisites of random sampling techniques and a general population sampling frame; in addition, we adjusted for methodological variability within the dataset through inclusion of several methodological covariates; however, methodological standards is an area which could be improved in future studies.

## Conclusion

This study provides a significant advancement on our current understanding of the epidemiology of PTSD and major depression in conflict-affected populations. It provides for the first time, age-specific estimates of PTSD and major depression prevalence drawing on the body of research undertaken within post-conflict populations and methods developed for GBD2013. The research was also able to examine a more extensive range of covariates that had been previously examined. The results provide important input (together with information on cost-effectiveness and equity) for estimating health service requirements. Future research should strengthen study methodology and design, including longitudinal assessments, and allow investigations of whether the risk of other mental disorders is elevated by conflict.

## Supplementary material

For supplementary material accompanying this paper visit http://dx.doi.org/10.1017/gmh.2015.26. click here to view supplementary material
