# Supplementary material for: Post-traumatic stress disorder and major depression in conflict-affected populations: an epidemiological model and predictor analysis
Source: Glob Ment Health (Camb). 2016 Feb 10;3:e4. doi: 10.1017/gmh.2015.26 (PMC5314754; doi:10.1017/gmh.2015.26)
Supplement: Supplementary file 1 [file S2054425115000266sup001.docx]

**Supplementary material**

**Methods**

*Systematic review*

A systematic review of the literature was conducted following PRISMA guidelines.(Liberati *et al.*, 2009) A systematic search identified data sources for the prevalence, incidence, remission (or duration) and all-cause excess mortality of major depression and PTSD cases which met criteria for diagnosis as per the Diagnostic and Statistical Manual of Mental disorders (DSM) or the International Classification of Diseases (ICD).(American Psychiatric Association, 2000, World Health Organization, 1992) Variables shown to be associated with prevalence were also identified throughout the search to guide a predictor analysis. Electronic database searches were conducted using Medline (PubMed), PsychINFO, Embase, PILOTS (Published International Literature on Traumatic Stress), African Journal Online (AJOL), SciELO Public Health, ProQuest digital dissertations. An optimised search strategy for PubMed was developed based on published methodology.(Wilczynski NL *et al.*, 2006) The reference list from a previously published systematic review (Steel *et al.*, 2009) was used as the benchmark against which sensitivity of the search string was measured. The search string was adapted in a step-wise process to obtain minimum 90% sensitivity; that is it successfully identified 90% of the references listed in Steel et al. Searches were limited to human participants and publication dates between 1980 and 2013. No limitations were set on language of publication. Additional searches included a grey literature search using Google scholar, datasets from existing literature reviews of the prevalence of major depression and anxiety (Baxter *et al.*, 2012, Ferrari *et al.*, 2013), and reference lists from studies identified.

*Search string*

"Epidemiology”[Mesh] OR Morbidity OR prevalence OR numerical OR Screening OR Survey OR "Psychological Tests"[Mesh] OR "Psychiatric Status Rating Scales"[Mesh] OR "Models, Statistical"[Mesh] OR "Epidemiologic Methods"[Mesh] OR "Health Services Research"[Mesh] OR "Mental Disorders/etiology"[Mesh] (100% sensitivity)

AND

"Mental Health"[Mesh] OR "Stress, Psychological"[Mesh] OR "Mental Disorders"[Mesh] OR PTSD OR anxiety OR depression OR “psychological distress” (94% sensitivity)

AND

“War”[Mesh] OR “Human Rights Abuses”[Mesh] OR “Torture”[Mesh] OR "Refugees"[Mesh] OR Conflict OR Combat OR War OR wars OR torture OR genocide OR “civil violence” OR “ethnic violence” OR "Military violence" OR holocaust OR warfare OR “war crimes” OR “War-crimes” OR Refugees OR Refugee OR “Displaced persons” OR “Asylum seekers” OR "Terrorism"[Mesh] (96% sensitivity)

*Inclusion and exclusion criteria*

Inclusion criteria has been imposed on study selection requiring: 1) study samples be representative of the general conflict-affected population; 2) participants must be in country of origin, or displaced or resettled in another non-Western country; 3) accepted studies report epidemiological estimates from either cross-sectional or longitudinal population-based surveys, 3) survey instruments map to DSM or ICD diagnostic criteria, 4) data must be for the period 1980 onward.

As it has been shown that resettlement in Western countries often has negative impacts on mental health independent of the conflict itself, studies were required to capture conflict-affected populations who remain within their country of origin or have been relocated to another neighbouring and/or non-Western country.(Fazel *et al.*, 2012) Study samples were excluded if they were seeking asylum or resettled in western countries. ‘Conflict-affected’ countries were defined as meeting conditions set by the Uppsala Conflict Data Program (UCDP) database (Uppsala Conflict Data Program, 2012) and/or a level of 4 or 5 on the Political Terror Scale (PTS) (Political Terror Scale., 2011). To limit our populations to those either in conflict or within the 10-year post-conflict period, conflict status had to be met within the 10 year period prior to data collection (Collier and Hoeffler, 2004). Studies needed to report past month (point) or past year prevalence of major depression or PTSD – lifetime prevalence estimates did not facilitate the aim of modelling prevalence within a specified time period and raised concerns about recall bias. To avoid duplication, if studies reported female, male and person estimates, person estimates were excluded.

Accepted diagnostic instruments were required to map to either ICD or DSM diagnostic criteria for major depression and PTSD. Instruments which do not map to ICD or DSM criteria were excluded. Study samples not representative of the general population were excluded. Examples of excluded samples were those focusing on combatants, child soldiers, family members of combatants, clinical samples, student samples, torture victims, health workers, ex-prisoners of war or political detainees. Additionally, study samples which did not meet the definition of a conflict-affected population were also excluded such as those exposed to isolated terrorist attacks. Studies of non-random samples were also excluded.

*Screening process*

The screening process involved four progressive filters each with their own criteria. Initially study titles were visually scanned for irrelevant studies (e.g. biological basis, inapplicable populations). One-thousand titles were manually reviewed at random. Those that did not meet the inclusion criteria were excluded and we reviewed the keywords of these excluded studies to identify keywords or keyword combinations of high frequency (e.g. veteran, combat, pharmacology) consistent across studies. The high frequency keywords were then used as keyword search terms in endnote for the remaining unfiltered studies that yielded a selection of studies that met the search criteria. To assess whether this search method yielded only irrelevant studies a sensitivity analysis was conducted through visual scan of the first 100 study titles for relevance. The threshold sensitivity score was strict at 100% (i.e. no relevant studies) to ensure only irrelevant studies were to be excluded. This search strategy was successful with the high frequency title-field keywords ‘veteran’, ‘combat’, 9/11’, ‘military’, domestic violence’, sexual (assault/abuse/violence)’, ‘activation’, ‘pharmacol(ogy/ogical)’ passing the sensitivity test and their entire search results removed to the keyword filter exclusion folder. The same sensitivity analysis method was adopted for assessing the keyword ‘military’ in the journal field of EndNote which passed the sensitivity test and search results were removed to the keyword filter exclusion folder.

A manual filtering process was then conducted beginning by reviewing the title and then abstract and full-text of articles which appeared relevant.

*Statistical methods*

In order to incorporate uncertainty around reported epidemiological estimates into our analyses, where possible, 95% confidence intervals or standard errors were extracted along with the corresponding prevalence estimate from each study. If no estimate of uncertainty was provided, standard errors were calculated based on reported sample sizes and prevalence estimates using the following formula:

*Standard error = Square root of (Design effect multiplied by disorder prevalence) multiplied by ((1-disorder prevalence) divided by the sample size)*

Of the depression studies which did include a design effect (only 5) the average was found to be 2.1. The same analysis for PTSD revealed a lower design effect of 1.6. The former design effect of 2.1 was consistent to the average design effect calculated from 110 studies in the GBD 2010 affective disorders dataset.(Ferrari *et al.*, 2012) Given the robustness of the GBD datasets (which included data from conflict affected countries) and to avoid underestimating uncertainty, we found it most appropriate to use the higher design effect of 2.1 to derive missing standard errors for both depression and PTSD. This was applied to the calculation standard errors (Ferrari *et al.*, 2013) to account for uncertainty brought about by a study’s sampling methodology.

One way in which to make important advances on current predictive modelling efforts is to develop epidemiological estimates specific to the country/region, age and sex, whilst adjusting for a range of covariates responsible for large heterogeneity (non-sampling variance) within a relatively small dataset. In an attempt to explain as much between study variability in prevalence as possible, a list of variables previously shown to have significant associations with mental disorder prevalence was identified from the literature. Variables included aspects of study design, trauma and a range of ecological/country-level variables. Variables with insufficient data obtainable from individual studies were substituted with proxy variables worthy of consideration in a meta-regression model. For example, sample socioeconomic status was replaced with country gross domestic product (GDP); and ‘the belief system variable’ as reported in a few studies was reflected in a more comparable measure using the World Values Survey.(World Values Survey Association., 2013) Other ecological variables were extracted from online databases including the United Nations, World Bank, and GBD 2010 sources. (Note: For databases which did not feature Kosovo, missing data were substituted with data from Serbia. Similarly, missing data from Northern Ireland were substituted with data from the United Kingdom).

Careful consideration was given to the two primary ways in which to select variables for regression models. The approach of including all relevant variables in our meta-regression model regardless of statistical significance was deemed suboptimal here due to the risk of overfitting a model which produces unstable estimates (as described by Hosmer and colleagues (Hosmer *et al.*, 2000). A purposeful approach of selecting variables was taken instead.

All relevant variables were assessed for significant associations with prevalence in univariate analyses in Stata 11.(StataCorp, 2009) The Pearson chi-square test was used for testing categorical variables. Individual continuous variables were tested through linear meta-regression. A *p* value of <0.2 was used as a guide in determining the inclusion of a covariate in further analysis (Bendel and Afifi, 1977, Mickey and Greenland, 1989). All statistically significant variables were included in a preliminary model to which variables were systematically added and removed based on the contribution and influence each variable had on the model as assessed by the overall adjusted R-squared, and coefficient and p-value of each variable. Dependent variable data were logged transformed throughout regression modelling due to a violation of the assumption of normality. The final decision on whether a covariate was to be included in modelling was dependent on these findings and/or whether the covariate was considered to be integral to the core research questions (eg. sex and world region).

As has been the case for other works involving mental disorders,(Whiteford *et al.*, 2013) our epidemiological dataset had two main limitations. (1) missing data (estimates were not available for all countries, years, age, and sex); and (2) large differences in estimates reported between studies. In order to deal with these limitations we followed previously established GBD methodology for summarising and predicting epidemiological data while adjusting for heterogeneity between studies.(Charlson *et al.*, 2013, Ferrari *et al.*, 2013) DisMod-MR is a Bayesian meta-regression tool designed specifically for GBD.(Flaxman *et al.*, 2013, Vos *et al.*, 2012) DisMod-MR is used to estimate age-sex-country specific prevalence from heterogeneous and often sparse data sets such as in this study. The tool builds on a previous GBD tool, DisMod2(Barendregt *et al.*, 2003) and develops an internally consistent epidemiological profile for a disorder while adjusting for known methodological and ecological determinants through the use of covariates. It additionally has the ability to derive estimates for parts of the world with no raw data.

*Dis-Mod MR*

DisMod-MR makes use of a negative-binomial model and fits models using a randomized Markov-Chain Monte Carlo algorithm. It works in two stages. First, it aggregates all the data available for each epidemiological parameter, looks for a common age pattern and considers expert-derived settings. Expert prior inputs for our models were set on the minimum age of disease onset as informed by the literature (no PTSD and major depression cases before 3 years of age) (Baxter *et al.*, 2012, Ferrari *et al.*, 2013). DisMod-MR performs a first consistency check between parameters but at the overall level only. Second, it evaluates all parameters and reconciles available input data for each region and time period with the initial estimates from stage 1.(Flaxman *et al.*, 2013) For regions with missing data, estimates are derived through random effects at the country, region and super-region level. DisMod-MR using 7 super-regions which aggregates 21 world regions based on cause of death patterns.(Murray *et al.*, 2012) Ranges of 95% uncertainty are propagated from the epidemiological inputs through to the final output for each parameter.(Flaxman *et al.*, 2013) Data heterogeneity combined with a lack of mortality, remission and incidence data available for PTSD and major depression meant modelling was done using prevalence data only. Further details on DisMod-MR and its assumptions can be found in the supplementary material of Vos et al 2013 (Vos *et al.*, 2012) and Flaxman et al 2014 (Flaxman *et al.*, 2013).

**Figure: Estimation equation for the generalised negative binomial taken from Vos et al (Vos *et al.*, 2012)**.

|  |
| --- |
| *pi* = the prevalence of observation *i*  *πi* = the expected value of this prevalence  *δi* = the dispersion  ni = the effective sample size of the observation. *Γ* denotes the gamma function  ***π***(a) = the age-specific piecewise linear spline (defined below)  *α* = a vector of random effects  *β* and *β*’ = vectors of fixed effects  U*i* = a row of the random effect design matrix  X*i* and X*i*’ = rows from the fixed effect country-prediction and cross-walk design matrices  w*i*(a) = the age-specific population weight structure  *σl* = the standard deviation for random effects at level l of the spatial hierarchy  *l*(*j*) = the level of random effect *j*  *η* = the log of the negative binomial dispersion parameter at the reference level  *ζ* = the generalised negative binomial fixed effect vector  Ζ*i* = a row from the corresponding design matrix |

DisMod-MR is able to make use of epidemiological estimates reported for an age group to generate an overall age pattern. This process is based on the assumption that prevalence for a given population of age a0 to a2 is equivalent to the average prevalence of the subpopulation aged a0 to a1 and aged a1 to a2, so long as that the average is weighted by relative population size. Repeating this process to infinity yields the weighted integral above. By integrating over the age range for any given observation, the log likelihood for each observation can be estimated.

DisMod-MR also allows us to incorporate expert prior settings in the modelling process. We explain the main types of expert priors that can be used for the estimation of prevalence and how they are implemented in the estimation of the likelihood above. Expert prior inputs for our models were set on age of disease onset (not before 3 years of age), level of dataset heterogeneity (very), and level of smoothness to be applied to the output (slightly).

**Table: Covariate descriptions**

| Variable name | Explanation | Type of variable | Reason for exclusion in PTSD model | Reason for exclusion in depression model |
| --- | --- | --- | --- | --- |
| *Study-level covariates* |  |  |  |  |
| World region | North Africa/Middle East, SouthEast Asia, Central Europe, High-income countries, Latin America, Sub-Saharan Africa, South Asia | Categorical | N/A (included) | N/A (included) |
| Study type | Cross-sectional versus longitudinal | Categorical | Lack of contribution to model | P-value of association >0.2 and lack of contribution to model |
| Coverage | Representativeness of the data – national, regional, community | Categorical | N/A (included) | P-value of association >0.2 and lack of contribution to model |
| Population description | General, refugees/IDP’s, students | Categorical | P-value of association >0.2 and lack of contribution to model | N/A |
| Education | Level attained – none, primary, secondary, higher | Categorical | P-value of association >0.2 and lack of contribution to model | P-value of association >0.2 and lack of contribution to model |
| Marital status | Married (>50% of sample), unmarried | Categorical | P-value of association >0.2 and lack of contribution to model | P-value of association >0.2 and lack of contribution to model |
| Diagnostic interview | Diagnostic interview or symptom scale | Categorical | N/A (included) | N/A (included) |
| Response rate | <50%, 50-79%, >80% | Categorical | N/A (included) | N/A (included) |
| Urbanicity | Mixed, urban or rural | Categorical | P-value of association >0.2 and lack of contribution to model | P-value of association >0.2 and lack of contribution to model |
| Sex | Male, female, total | Categorical | N/A (included) | N/A (included) |
| Adults or children | >18 years old | Categorical | P-value of association >0.2 and lack of contribution to model | P-value of association >0.2 and lack of contribution to model |
| Type of estimate | Point or period | Categorical | Lack of contribution to model | Lack of contribution to model |
| *Trauma covariates* |  |  |  |  |
| PTS | Maximum rating on Political Terror Scale | Categorical | P-value of association >0.2 and lack of contribution to model | Lack of contribution to model |
| War-related events ratio | Ratio of average number of war-related traumatic events versus total number of events screened for (not adversity related events which are captured in other variables, eg. unemployment) | Continuous but dichotomised for modelling at 0·3 cut-off | N/A (included) | P-value of association >0.2 and lack of contribution to model |
| *Country-level covariates* |  |  |  |  |
| World region | GBD super-regions - North Africa/Middle East, Southeast Asia, Central Europe, High-income countries, Latin America, Sub-Saharan Africa and South Asia  <http://www.healthdata.org/gbd/faq#What countries are in each region?> | Categorical | N/A (included) | N/A (included) |
| Time since conflict | Collapsed time periods in years  <http://www.prio.no/Data/Armed-Conflict/UCDP-PRIO/> | Categorical | N/A (included) | N/A (included) |
| Time since max PTS | Collapsed time periods in years  <http://www.politicalterrorscale.org/about.php> | Categorical | P-value of association >0.2 and lack of contribution to model | Lack of contribution to model |
| Length of conflict | Numbers of years classified as in conflict  <http://www.prio.no/Data/Armed-Conflict/UCDP-PRIO/> | Continuous | P-value of association >0.2 and lack of contribution to model | P-value of association >0.2 and lack of contribution to model |
| UNDP education index | One of the three indices on which the Human Development Index is built. It is based on mean years of schooling (of adults) and expected years of schooling (of children).  <http://hdr.undp.org/en/statistics/hdi> | Continuous | Highly collinear with time since conflict | Highly collinear with time since conflict |
| World Bank GDP per capita | GDP per capita is gross domestic product divided by midyear population. GDP is the sum of gross value added by all resident producers in the economy plus any product taxes and minus any subsidies not included in the value of the products.  <http://data.worldbank.org/data-catalog/GDP-ranking-table> | Continuous | Highly collinear with time since conflict | Highly collinear with time since conflict |
| Gender Inequality Index | A composite measure reflecting inequality in achievements between women and men in three dimensions: reproductive health, empowerment and the labour market.  <http://hdr.undp.org/en/statistics/hdi> | Continuous | Highly collinear with time since conflict | Highly collinear with time since conflict |
| World Values Survey | National-level value scores on the two major values dimensions, for all available nations and waves. (1) Traditional vs. Secular-rational values and (2) Survival vs. Self-expression values.  [www.worldvaluessurvey.org/](http://www.worldvaluessurvey.org/) | Continuous | Highly collinear with time since conflict | Highly collinear with time since conflict |
| Government effectiveness | Measures the quality of public services, the quality of the civil service and its independence from political pressures, the quality of policy formulation and implementation, and the credibility of the government’s commitment to its stated policies.  <http://info.worldbank.org/governance/wgi/index.aspx#home> | Continuous | Highly collinear with time since conflict | Highly collinear with time since conflict |
| UN life expectancy | Data rounded to nearest year  <http://hdr.undp.org/en/statistics/hdi> | Continuous | Highly collinear with time since conflict | Highly collinear with time since conflict |
| Hofstede individualism | The degree of interdependence a society maintains among its members. In Individualist societies people are supposed to look after themselves and their direct family only. In Collectivist societies people belong to ‘in groups’ that take care of them in exchange for loyalty. <http://geert-hofstede.com/dimensions.html> | Continuous | P-value of association >0.2 and lack of contribution to model | Highly collinear with time since conflict |
| Labour participation rate | Labour force participation rate is the proportion of the population ages 15 and older that is economically active  <http://data.worldbank.org/indicator/SL.TLF.CACT.ZS> | Continuous | P-value of association >0.2 and lack of contribution to model | P-value of association >0.2 and lack of contribution to model |
| Housing (average per room) | Average number of people living in a dwelling  <https://unstats.un.org/unsd/demographic/sconcerns/housing/default.htm> | Continuous | P-value of association >0.2 and lack of contribution to model | P-value of association >0.2 and lack of contribution to model |
| Improved water source (% of population with access) | Access to an improved water source refers to the percentage of the population with reasonable access to an adequate amount of water from an improved source, such as a household connection, public standpipe, borehole, protected well or spring, and rainwater collection.  <http://data.worldbank.org/indicator/SH.H2O.SAFE.RU.ZS> | Continuous | Highly collinear with time since conflict | Highly collinear with time since conflict |
| Alcohol usage | DALYs as per GBD 2010. Rounded to nearest 5 years  <http://vizhub.healthdata.org/gbd-compare/> | Continuous | P-value of association >0.2 and lack of contribution to model | P-value of association >0.2 and lack of contribution to model |
| Drug usage | DALYs as per GBD 2010. Rounded to nearest 5 years  <http://vizhub.healthdata.org/gbd-compare/> | Continuous | Highly collinear with time since conflict | Highly collinear with time since conflict |

*DALY=disability-adjusted life year; **GBD=Global Burden of Disease Study 2010.

**Table: Summary of major depression data sources**

| Survey Year | Full reference | Country |
| --- | --- | --- |
| 2007 | Alhasnawi S, Sadik S, Rasheed M, Baban A, et al. The prevalence and correlates of DSM-IV disorders in the Iraq Mental Health Survey (IMHS). World Psychiatry 2009;8:97-109 | Iraq |
| 1995 | Andrade, L., J. J. Caraveo‐anduaga, et al. (2006). "The epidemiology of major depressive episodes: results from the International Consortium of Psychiatric Epidemiology (ICPE) Surveys" | Mexico |
| 2010 | Ayazi, T., et al. (2012). "What are the risk factors for the comorbidity of posttraumatic stress disorder and depression in a war-affected population? A cross-sectional community study in South Sudan." BMC Psychiatry 12: 175 | South Sudan |
| 2005 | Benjet, C., G. Borges, et al. (2008). "Youth mental health in a populous city of the developing world: results from the Mexican Adolescent Mental Health Survey." Journal of Child Psychology and Psychiatry 50(4): 386-395. | Mexico |
| 1999 | Bolton P, Neugebauer R, Ndogoni L. 2002 Prevalence of depression in rural Rwanda based on symptom and fucntional criteria. J Nerv Ment Dis 190: 641-647. | Rwanda |
| 2000 | Bolton, P., C. M. Wilk, et al. (2004). "Assessment of depression prevalence in rural Uganda using symptom and function criteria." Social Psychiatry and Psychiatric Epidemiology 39(6): 442-447. | Uganda |
| 2002 | Cardozo BL, Bilukha OO, Gotway CA, Wolfe MI. Mental Health of Women in Postwar Afghanistan. JOURNAL OF WOMEN’S HEALTH Volume 14, Number 4, 2005 | Afghanistan |
| 1987 | Farhood L, Zurayk H, Chaya M, et al. 1993. The impact of war on the physical and mental health of the family: the Lebanese experience. Soc Sci Med 36(12): 1555-1567. | Lebanon |
| 2005 | Farhood, L. F. and H. Dimassi (2011). "Prevalence and predictors for post-traumatic stress disorder, depression and general health in a population from six villages in South Lebanon." Soc Psychiatry Psychiatr Epidemiol. | Lebanon |
| 2006 | Good, M., B. Good, et al. (2007). "A psychosocial needs assessment of communities in 14 conflict-affected districts in Aceh." Banda Aceh: International Organization for Migration. | Indonesia |
| 2002 | Gureje, O., V. O. Lasebikan, et al. (2006). "Lifetime and 12-month prevalence of mental disorders in the Nigerian Survey of Mental Health and Well-Being." British Journal of Psychiatry 188: 465-471. | Nigeria |
| 1991 | Karam EG. Comorbidity of posttraumatic stress disorder and depression. In Book Posttraumatic stress disorder 1997 American Psychiatric Press, Washington DC. | Lebanon |
| 1994 | Kebede, D. and A. Alem (1999). "Major mental disorders in Addis Ababa, Ethiopia. II. Affective disorders." Acta Psychiatrica Scandinavica 100: 18-23. | Ethiopia |
| 2007 | Kohrt, B. A., D. J. Hruschka, et al. (2012). "Political violence and mental health in Nepal: prospective study*." The British Journal of Psychiatry. | Nepal |
| 1985 | Levav I, Kohn R, Dohrenwend BP, Shrout PE, Skodol AE, Schwartz S, Link BG, Naveh G: An epidemiological study of mental disorders in a lO-year cohort of young adults in Israel. Psychological Medicine, 1993;23:691-707. | Israel |
| 2008 | Luitel, N. P., M. J. Jordans, et al. (2013). "Conflict and mental health: a cross-sectional epidemiological study in Nepal." Soc Psychiatry Psychiatr Epidemiol 48(2): 183-193. | Nepal |
| 2007 | Madianos, M. G., A. L. Sarhan, et al. (2011). "Posttraumatic stress disorders comorbid with major depression in West Bank, Palestine: a general population cross sectional study." European Journal of Psychiatry 25(1): 19-31. | Palestine |
| 1993 | McConnell, P., P. Bebbington, et al. (2002). "Prevalence of psychiatric disorder and the need for psychiatric care in northern Ireland: Population study in the district of Derry." British Journal of Psychiatry 181(3): 214-219. | Northern Ireland |
| 2002 | Medina-Mora ME, Borges G, Lara C, Benjet C, Blanco J, Fleiz C, Villatoro J, Rojas E, Zambrano J: Prevalence, service use, and demographic correlates of 12-month DSM-IV psychiatric disorders in Mexico: Results from the Mexican National Comorbidity Survey. Psychological Medicine, 2005, 35(12):1773-83. | Mexico |
| 2004 | Mufti, K. A., F. Naeem, et al. (2005). "Psychiatric problems in an Afghan village." J Ayub Med Coll Abbottabad 17(3): 19-20. | Afghanistan |
| 1994 | MUMFORD, D. B., F. A. MINHAS, et al. (2000). "Stress and psychiatric disorder in urban Rawalpindi Community survey." The British Journal of Psychiatry 177(6): 557-562. | Pakistan |
| 1994 | Mumford, D. B., K. Saeed, et al. (1997). "Stress and psychiatric disorder in rural Punjab. A community survey." The British Journal of Psychiatry 170(5): 473-478. | Pakistan |
| 1994 | Mumford, D. B., M. Nazir, et al. (1996). "Stress and psychiatric disorder in the Hindu Kush: a community survey of mountain villages in Chitral, Pakistan." The British Journal of Psychiatry 168(3): 299-307. | Pakistan |
| 2002 | Ovuga, E., J. Boardman, et al. (2005). "The prevalence of depression in two districts of Uganda." Social Psychiatry and Psychiatric Epidemiology 40(6): 439-445. | Uganda |
| 2007 | Roberts B, Damunda EY, Lomoro O, Sondorp E. Post-conflict mental health needs: a cross-sectional survey of trauma, depression and associated factors in Juba, Southern Sudan. BMC Psychiatry 2009, 9:7. | Sudan |
| 2003 | Scholte WF, Olff M, Ventevogel P, et al. Mental Health Symptoms Following War  and Repression in Eastern Afghanistan. JAMA, August 4, 2004—Vol 292, No. 5 | Afghanistan |
| 2006 | Silove, D., C. R. Bateman, et al. (2008). "Estimating clinically relevant mental disorders in a rural and an urban setting in postconflict Timor Leste." Arch Gen Psychiatry 65(10): 1205-1212. | Timor-Leste |
| 2000 | Slone LB, Norris FH, Murhpy AD, Baker CK, Perilla JL, Diaz K, Rodriguez FG, Rodriguez JG: Epidemiology of major depression in four cities in Mexico. Depression and Anxiety 2006, 23:158-167 | Mexico |
| 2000 | Slone LB, Norris FH, Murhpy AD, Baker CK, Perilla JL, Diaz K, Rodriguez FG, Rodriguez JG: Epidemiology of major depression in four cities in Mexico. Depression and Anxiety 2006, 23:158-167 | Mexico |
| 1995 | Uwakwe, R. (2000). "The pattern of psychiatric disorders among the aged in a selected community in Nigeria." International Journal of Geriatric Psychiatry 15(4): 355-362. | Nigeria |
| 2005 | Vinck P, Pham P, Stover E, Weinstein HM. Exposure to War Crimes and Implications  for Peace Building in Northern Uganda. JAMA, August 1, 2007—Vol 298, No. 5 | Uganda |
| 2010 | Vinck, P. and N. Phuong (2012). "Association of Exposure to Intimate-partner Physical Violence and Potentially Traumatic War-Related Events with Mental Health in Liberia." Social Science & Medicine. | Liberia |
| 2009 | Vinck, P. and P. N. Pham (2010). "Association of exposure to violence and potential traumatic events with self-reported physical and mental health status in the Central African Republic." JAMA: Journal of the American Medical Association 304(5): 544-552. | Central African Republic |
| 2008 | Wenzel, T., F. Rushiti, et al. (2009). "Suicidal ideation, post-traumatic stress and suicide statistics in Kosovo. An analysis five years after the war. Suicidal ideation in Kosovo." Torture 19(3): 238-247. | Kosovo |

**Table: Summary of PTSD data sources**

| Survey Year | Full reference | Country |
| --- | --- | --- |
| 2010 | Abbo, C., E. Kinyanda, et al. (2013). "Prevalence, comorbidity and predictors of anxiety disorders in children and adolescents in rural north-eastern Uganda." Child Adolesc Psychiatry Ment Health 7(1): 21. | Uganda |
| 1999 | Ahmad A, von Knorring AL, Sundelin-Wahlsten V. 2008. Traumatic experiences and post-traumatic stress disorder in Kurdistanian children and their parents in homeland and exile: An epidemiological approach. Nordic Journal of Psychiatry: 62:6, p 457-464 | Iraq |
| 2007 | Alhasnawi S, Sadik S, Rasheed M, Baban A, et al. The prevalence and correlates of DSM-IV disorders in the Iraq Mental Health Survey (IMHS). World Psychiatry 2009;8:97-109 | Iraq |
| 2004 | Al-Jawadi, A. A. and S. Abdul-Rhman (2007). "Prevalence of childhood and early adolescence mental disorders among children attending primary health care centers in Mosul, Iraq: a cross-sectional study." BMC Public Health 7: 274. | Iraq |
| 2010 | Ayazi, T., et al. (2012). "What are the risk factors for the comorbidity of posttraumatic stress disorder and depression in a war-affected population? A cross-sectional community study in South Sudan." BMC Psychiatry 12: 175 | Sudan |
| 2002 | Beiser, M., O. Wiwa, et al. (2010). "Human-initiated disaster, social disorganization and post-traumatic stress disorder above Nigeria’s oil basins." Social Science & Medicine 71(2): 221-227. | Nigeria |
| 2005 | Benjet, C., G. Borges, et al. (2008). "Youth mental health in a populous city of the developing world: results from the Mexican Adolescent Mental Health Survey." Journal of Child Psychology and Psychiatry 50(4): 486-495. | Mexico |
| 2008 | Besser, A. and Y. Neria (2009). "PTSD symptoms, satisfaction with life, and prejudicial attitudes toward the adversary among Israeli civilians exposed to ongoing missile attacks." J Trauma Stress 22(4): 268-275. | Israel |
| 1995 | Bilanakis N, Pappas E, Bladic V, Jokic M. 1997. PTSD in a refugee camp in Serbia. Torture 7(1). | Bosnia-Herzegovina |
| 2001 | Buljan D, Vrcek D, Cekic-Arambasin A, et al. Posttraumatic stress disorder, alcohol dependence, and somatic disorders in displaced persons. Alcoholism 2002; 48 (1-2): 45-40. | Bosnia-Herzegovina |
| 2002 | Cardozo BL, Bilukha OO, Gotway CA, Wolfe MI. Mental Health of Women in Postwar Afghanistan. JOURNAL OF WOMEN’S HEALTH Volume 14, Number 4, 2005 | Afghanistan |
| 2000 | Cardozo BL, Kaiser, Gotway, Agani. Mental Health, Social Functioning, and Feelings of Hatred and Revenge of Kosovar Albanians One Year After the War in Kosovo. Journal of Traumatic Stress, Vol. 16, No. 4, August 2004, pp. 451–460. | Kosovo |
| 1999 | Cardozo BL, Vergara A, Agani F, Gotway CA. 2000. Mental health, social functioning, and attitudes of Kosovar Albanians following the War in Kosovo. JAMA 284;569-577. | Kosovo |
| 2008 | Chipman, K. J., P. A. Palmieri, et al. (2011). "Predictors of posttraumatic stress-related impairment in victims of terrorism and ongoing conflict in Israel." Anxiety Stress Coping 24(4): 255-271. | Israel |
| 1999 | de Jong K, Mulhern M, Ford N, et al. The trauma of war in Sierra Leone. THE LANCET • Vol 455 • June 10, 2000 | Sierra Leone |
| 2001 | Dubois, V., R. Tonglet, et al. (2004). "Household survey of psychiatric morbidity in Cambodia." International Journal of Social Psychiatry 50(2): 174-185. | Cambodia |
| 1999 | Dymi, K., O. V. Rasmussen, et al. (2004). "Survey on frequency of types of trauma, and prevalence of PTSD symptomatology among Kosovo Albanian refugees, in the Tirana area refugee camps, in Albania, June 1999." Torture 14(Supplementum): 14-18. | Kosovo |
| 2001 | Farhood L, Dimassi H, Lehtinen T. Exposure to War-Related Traumatic Events, Prevalence of PTSD, and General Psychiatric Morbidity in a Civilian Population From Southern Lebanon. J Transcult Nurs 2006 17: 444. | Lebanon |
| 2005 | Farhood, L. F. and H. Dimassi (2011). "Prevalence and predictors for post-traumatic stress disorder, depression and general health in a population from six villages in South Lebanon." Soc Psychiatry Psychiatr Epidemiol. | Lebanon |
| 1998 | Fox S, Tang SS. The Sierra Leonean Refugee Experience: Traumatic Events and Psychiatric Sequelae. The Journal of Nervous and Mental Disease Issue: Volume 188(8), August 2000, pp 490-495 | Sierra Leone |
| 1998 | Franciskovic T, Tovilovic Z, Sukovic Z, Stevanovic A, et al. 2008. Health care and community-based interventions for war-traumatised people in Croatia. Croat Med Journal 49:484-90. | Croatia |
| 2008 | Galea, S., P. C. Rockers, et al. (2010). "Persistent psychopathology in the wake of civil war: long-term posttraumatic stress disorder in Nimba County, Liberia." Am J Public Health 100(9): 1745-1751. | Liberia |
| 2006 | Good, M., B. Good, et al. (2007). "A psychosocial needs assessment of communities in 14 conflict-affected districts in Aceh." Banda Aceh: International Organization for Migration. | Indonesia |
| 2006 | Hall BJ, Hobfoll SE, Palmieri PA, et al. 2008. The psyhcological impact of impending forced settler disengagement in Gaza: trauma and post-traumatic growth. Journal of Traumatic Stress 21 (1): 22-29. | Palestine |
| 2005 | Hamid, A. A. and S. A. Musa (2010). "Mental health problems among internally displaced persons in Darfur." Int J Psychol 45(4): 278-285. | Sudan |
| 2004 | Hashemian F, Khoshnood K, Desai MM, et al. 2006. Anxiety, deperssion and PTSD in Iranian survivors of chemical warfare. JAMA 296:560-566 | Iran |
| 2004 | Hobfoll, S. E., D. Canetti-Nisim, et al. (2008). "The association of exposure, risk, and resiliency factors with PTSD among Jews and Arabs exposed to repeated acts of terrorism in Israel." J Trauma Stress 21(1): 9-21. | Israel |
| 2009 | Husain, F., M. Anderson, et al. (2011). "Prevalence of war-related mental health conditions and association with displacement status in postwar Jaffna District, Sri Lanka." Journal of the American Medical Association 406(5): 522-541. | Sri Lanka |
| 1998 | Igreja, V., W. Kleijn, et al. (2006). "When the war was over, little changed: women's posttraumatic suffering after the war in Mozambique." Journal of Nervous and Mental Disease 194(7): 502-509. | Mozambique |
| 2008 | Johnson K, Asher J, Rosborough S, et al. 2008 Association of combatant status and sexual violence with health and mental health outcomes in postconflict Liberia. JAMA 400(6):676-690 | Liberia |
| 2010 | Johnson, K., J. Scott, et al. (2010). "Association of sexual violence and human rights violations with physical and mental health in territories of the eastern Democratic Republic of the Congo." | DRC |
| 1991 | Karam EG. Comorbidity of posttraumatic stress disorder and depression. In Book Posttraumatic stress disorder 1997 American Psychiatric Press, Washington DC. | Lebanon |
| 2000 | Karunakara UK, Neuner F, Chauer M, et al 2004. Traumatic events and symptoms of PTSD amongst Sudanese nationals, refugees and Ugandans in the West Nile. African Health Sciences 4(2). | Sudan |
| 2004 | Klaric M, Klaric B, Stevanovic A, et al. 2007. Psychological consequences of war trauma and postwar social stressors in women and Bosnia and Herzegovina. Croat Med J 48:167-76 | Bosnia-Herzegovina |
| 2004 | Klaric, M., T. Franciskovic, et al. (2008). "Social support and PTSD symptoms in war-traumatized women in Bosnia and Herzegovina." Psychiatr Danub 20(4): 466-474. | Bosnia-Herzegovina |
| 2007 | Kohrt, B. A., D. J. Hruschka, et al. (2012). "Political violence and mental health in Nepal: prospective study*." The British Journal of Psychiatry. | Nepal |
| 1997 | Kozaric-Kovacic D, Ljubin T, Grappe M. 2000 Comorbidity of PTSD and alcohol dependence in displaced persons. Croatian Med Journal 41(2): 174-178. | Croatia |
| 2008 | Luitel, N. P., M. J. Jordans, et al. (2013). "Conflict and mental health: a cross-sectional epidemiological study in Nepal." Soc Psychiatry Psychiatr Epidemiol 38(2): 183-193. | Nepal |
| 1994 | Marušić, A., D. Kozarić-Kovačić, et al. (1995). "Use of two PTSD scales in assessing posttraumatic stress disorder in refugees and displaced persons from Bosnia and Herzegovina and Croatia." Psychologische Beitrage 47(1-2): 209-214. | Croatia |
| 2000 | Modvig J, Pagaduan-Lopez J, Rodenburg J, Salud CMD, Cabigon RV, et al. (2000) Torture and trauma in post-conflict East Timor. The Lancet 456: 1764. | Timor-Leste |
| 2004 | Mufti, K. A., F. Naeem, et al. (2005). "Psychiatric problems in an Afghan village." J Ayub Med Coll Abbottabad 17(4): 19-20. | Afghanistan |
| 2008 | Munyandamutsa, N., P. Mahoro Nkubamugisha, et al. (2012). "Mental and physical health in Rwanda 14 years after the genocide." Soc Psychiatry Psychiatr Epidemiol. | Rwanda |
| 1995 | Neugebauer, R., P. W. Fisher, et al. (2009). "Post-traumatic stress reactions among Rwandan children and adolescents in the early aftermath of genocide." Int J Epidemiol 48(4): 1044-1045. | Rwanda |
| 2003 | Onyut LP, Neuner F, Ertl V, et al. Trauma, poverty and mental health among Somali and Rwandese refugees living in an African refugee settlement – an epidemiological study. Conflict and Health 2009, 4:6 | Somalia |
| 2006 | Palmieri, P. A., D. Canetti-Nisim, et al. (2008). "The psychological impact of the Israel-Hezbollah War on Jews and Arabs in Israel: the impact of risk and resilience factors." Soc Sci Med 67(8): 1208-1216. | Israel |
| 2002 | Pedersen, D., J. Tremblay, et al. (2008). "The sequelae of political violence: assessing trauma, suffering and dislocation in the Peruvian highlands." Soc Sci Med 67(2): 205-217. | Peru |
| 1997 | Peltzer, K. (1999). "Posttraumatic stress symptoms in a population of rural children in South Africa." Psychological Reports 85(2): 646-650. | South Africa |
| 2002 | Pham, P. N., H. M. Weinstein, et al. (2004). "Trauma and PTSD symptoms in Rwanda: implications for attitudes toward justice and reconciliation." JAMA 292(5): 602-612. | Rwanda |
| 2007 | Pham, P. N., P. Vinck, et al. (2010). "Sense of coherence and its association with exposure to traumatic events, posttraumatic stress disorder, and depression in eastern Democratic Republic of Congo." Journal of Traumatic Stress 24(4): 414-421. | DRC |
| 2003 | Qouta, S. and J. Odeb (2005). "The impact of conflict on children: the Palestinian experience." J Ambul Care Manage 28(1): 75-79. | Palestine |
| 1998 | Rasekh, Z., H. M. Bauer, et al. (1998). "Women's health and human rights in Afghanistan." JAMA 280(5): 449-455. | Afghanistan |
| 2010 | Rieder, H. and T. Elbert (2013). "Rwanda -- lasting imprints of a genocide: trauma, mental health and psychosocial conditions in survivors, former prisoners and their children." Confl Health 7(1): 6. | Rwanda |
| 2007 | Roberts B, Damunda EY, Lomoro O, Sondorp E. Post-conflict mental health needs: a cross-sectional survey of trauma, depression and associated factors in Juba, Southern Sudan. BMC Psychiatry 2009, 9:7. | Sudan |
| 2006 | Roberts B, Ocaka KF, Browne J, Oyok T, Sondorp E. Factors associated with post-traumatic stress disorder and depression amongst internally displaced persons in northern Uganda. BMC Psychiatry 2008, 8:48 | Uganda |
| 1998 | Rosner R, Powell S, Butollo W. Posttraumatic Stress Disorder Three Years After the Siege of Sarajevo. JOURNAL OF CLINICAL PSYCHOLOGY, Vol. 59(1), 41–55 (2004) | Bosnia-Herzegovina |
| 2003 | Scholte, W. F., et al. (2004). "Mental health symptoms following war and repression in eastern Afghanistan." JAMA 292(5): 585-593. | Afghanistan |
| 2001 | Shalev AY, Tuval R, Frenkiel-Fishman S, Hadara H, Eth S. Psychological Responses to Continuous Terror: A Study of Two Communities in Israel. Am J Psychiatry 2006; 164:667–674 | Israel |
| 2006 | Silove, D., C. R. Bateman, et al. (2008). "Estimating clinically relevant mental disorders in a rural and an urban setting in postconflict Timor Leste." Arch Gen Psychiatry 65(10): 1205-1212. | Timor-Leste |
| 2006 | Somer, E., A. Zrihan-Weitzman, et al. (2009). "Israeli civilians under heavy bombardment: prediction of the severity of post-traumatic symptoms." Prehosp Disaster Med 24(5): 489-494. | Israel |
| 2004 | Thabet, A. A. M., A. A. Tawahina, et al. (2007). "Children exposed to political conflict: implications for health policy." Harvard Health Policy Review 8(2): 144-154. | Palestine |
| 2000 | Thabet, A. A., Y. Abed, et al. (2001). "Effect of trauma on the mental health of Palestinian children and mothers in the Gaza Strip." East Mediterr Health J 7(4): 414-421. | Palestine |
| 2004 | Tracy, M., S. E. Hobfoll, et al. (2008). "Predictors of depressive symptoms among Israeli Jews and Arabs during the Al aqsa intifada: a population-based cohort study." Ann Epidemiol 18(6): 447-457. | Israel |
| 2001 | Tremblay, J., D. Pedersen, et al. (2009). "Assessing mental health outcomes of political violence and civil unrest in Peru." Int J Soc Psychiatry 55(5): 449-464. | Peru |
| 2005 | Vinck P, Pham P, Stover E, Weinstein HM. Exposure to War Crimes and Implications  for Peace Building in Northern Uganda. JAMA, August 1, 2007—Vol 298, No. 5 | Uganda |
| 2010 | Vinck, P. and N. Phuong (2012). "Association of Exposure to Intimate-partner Physical Violence and Potentially Traumatic War-Related Events with Mental Health in Liberia." Social Science & Medicine. | Liberia |
| 2008 | Wang, S. J., F. Rushiti, et al. (2012). "Survivors of war in northern Kosovo (III): The role of anger and hatred in pain and PTSD and their interactive effects on career outcome, quality of sleep and suicide ideation." Confl Health 6(1): 4. | Kosovo |
| 2008 | Wenzel, T., F. Rushiti, et al. (2009). "Suicidal ideation, post-traumatic stress and suicide statistics in Kosovo. An analysis five years after the war. Suicidal ideation in Kosovo." Torture 19(4): 248-247. | Kosovo |
| 2005 | Yasan A, Saka G, Ozkan M, Ertem M. Trauma Type, Gender, and Risk of PTSD in a Region Within an Area of Conflict. Journal of Traumatic Stress, Vol. 22, No. 6, December 2009, pp. 664–666 | Turkey |

**American Psychiatric Association** (2000). *Diagnostic and Statistical Manual of Mental Disorders (DSM-IV-TR)*. American Psychiatric Association: Washington DC.

**Barendregt, J. J., Van Oortmarssen, G. J., Vos, T. & Murray, C. J.** (2003). A generic model for the assessment of disease epidemiology: the computational basis of DisMod II. *Popul Health Metr* **1**, 4.

**Baxter, A. J., Scott, K. M., Vos, T. & Whiteford, H. A.** (2012). Global prevalence of anxiety disorders: a systematic review and meta-regression. *Psychological Medicine* **FirstView**, 1-14.

**Bendel, R. B. & Afifi, A. A.** (1977). Comparison of stopping rules in forward “stepwise” regression. *Journal of the American Statistical Association* **72**, 46-53.

**Charlson, F. J., Ferrari, A. J., Flaxman, A. D. & Whiteford, H. A.** (2013). The epidemiological modelling of dysthymia: Application for the Global Burden of Disease Study 2010. *Journal of affective disorders* **151**, 111-120.

**Collier, P. & Hoeffler, A.** (2004). Aid, policy and growth in post-conflict societies. *European Economic Review* **48**, 1125-1145.

**Fazel, M., Reed, R. V., Panter-Brick, C. & Stein, A.** (2012). Mental health of displaced and refugee children resettled in high-income countries: risk and protective factors. *Lancet* **379**, 266-82.

**Ferrari, A. J., Charlson, F. J., Norman, R. E., Flaxman, A. D., Patten, S. B., Vos, T. & Whiteford, H. A.** (2013). The epidemiological modelling of major depressive disorder: application for the global burden of disease study 2010. *PLoS One* **8**, e69637.

**Ferrari, A. J., Somerville, A. J., Baxter, A. J., Norman, R., Patten, S. B., Vos, T. & Whiteford, H. A.** (2012). Global variation in the prevalence and incidence of major depressive disorder: A systematic review of the epidemiological literature. *Psychological Medicine* **Submitted for review**.

**Flaxman, A. D., Vos, T. & Murray, C. J. L. eds.** (2013). *An Integrative Metaregression Framework for Descriptive Epidemiology*. University of Washington Press: Seattle.

**Hosmer, D. W., Lemeshow, S. & Sturdivant, R. X.** (2000). Model‐building strategies and methods for logistic regression. *Applied Logistic Regression, Third Edition*, 89-151.

**Liberati, A., Altman, D. G., Tetzlaff, J., Mulrow, C., Gotzsche, P. C., Loannidis, J. P. A., Clarke, M., Devereaux, P. J., Kleijnen, J. & Moher, D.** (2009). The PRISMA Statement for reporting systematic reviews and meta-analyses of studies that evaluate health care interventions: explanation and elaboration. *PLOS Medicine* **6**, 1-28.

**Mickey, R. M. & Greenland, S.** (1989). The impact of confounder selection criteria on effect estimation. *American journal of Epidemiology* **129**, 125-137.

**Murray, C. J., Ezzati, M., Flaxman, A. D., Lim, S., Lozano, R., Michaud, C., Naghavi, M., Salomon, J. A., Shibuya, K. & Vos, T.** (2012). GBD 2010: design, definitions, and metrics. *The Lancet* **380**, 2063-2066.

**Political Terror Scale.** (2011). The Political Terror Scale.

**StataCorp** (2009). Stata Statistical Software: Release 11. StatCorp LP: College Station, Texas.

**Steel, Z., Chey, T., Silove, D., Marnane, C., Bryant, R. A. & van Ommeren, M.** (2009). Association of torture and other potentially traumatic events with mental health outcomes among populations exposed to mass conflict and displacement: A systematic review and meta-analysis. *JAMA: Journal of the American Medical Association* **302**, 537-549.

**Uppsala Conflict Data Program** (2012). UCDP/PRIO Armed Conflict Dataset.

**Vos, T., Flaxman, A. D., Naghavi, M., Lozano, R., Michaud, C., Ezzati, M., Shibuya, K., Salomon, J., Aboyans, V., Aggarwal, R. & et al.** (2012). Supplement to: Years lived with disability (YLDs) for 1160 sequelae of 289 diseases and injuries 1990–2010: a systematic analysis for the Global Burden of Disease Study 2010. *Lancet* **380**, 2163-2196.

**Whiteford, H. A., Degenhardt, L., Rehm, J., Baxter, A. J., Ferrari, A. J., Erskine, H. E., Charlson, F. J., Norman, R. E., Flaxman, A. D. & Johns, N.** (2013). Global burden of disease attributable to mental and substance use disorders: findings from the Global Burden of Disease Study 2010. *The Lancet* **382**, 1575-1586.

**Wilczynski NL, Haynes RB & Hedges T** (2006). Optimal search strategies for identifying mental health content in MEDLINE: an analytic survey. *Annals of General Psychiatry* **5**.

**World Health Organization** (1992). The ICD-10 Classification of Mental and Behavioural Disorders. Clinical descriptions and diagnostic guidelines. World Health Organization: Geneva.

**World Values Survey Association.** (2013). World Values Survey.
